# Major depression and mortality from cancer: A 10-year Italian study

**DOI:** 10.1192/j.eurpsy.2025.10048

**Published:** 2025-12-04

**Authors:** Luigi Grassi, Elisa Stivanello, Martino Belvederi Murri, Marco Cruciata, Angela Muscettola, Vincenza Perlangeli, Paolo Pandolfi, Barbara Zaccagnino, Elena Meggiolaro, Federica Ruffilli, Marco Menchetti, Ilaria Panzini, Domenico Berardi, Rosangela Caruso

**Affiliations:** 1 Institute of Psychiatry, Department of Neuroscience and Rehabilitation, University of Ferrara, Ferrara, Italy; 2 Integrated Department of Mental Health and Addictive Disorders, Local Health Trust and S’Anna University Hospital of Ferrara, Italy; 3 Department of Public of Health, Local Health Trust of Bologna, Bologna, Italy; 4 Department of Mental Health, ASL, Tivoli, Roma; 5 IRCCS Istituto Romagnolo Per Lo Studio Dei Tumori “Dino Amadori” – IRST S.R.L., Meldola, Italy; 6 Department of Biomedical and Neuromotor Sciences, University of Bologna, Bologna, Italy; 7 Unit of Research and Innovation, Local Health Trust of Ferrara, Ferrara, Italy

**Keywords:** cancer, major depression, mortality, oncology, psycho-oncology

## Abstract

**Background:**

To prospectively examine cancer-related mortality over a 10-year period in patients affected by major depression (MDD), Emilia Romagna Region (ERR), Northern Italy, and to compare these findings with those observed in the regional population.

**Methods:**

The ERR Mental Health Registry was used to identify all patients aged ≥18 years who accessed the ERR mental health departments with an ICD-9CM diagnosis of MDD between 2008–2017. Information on deaths were retrieved from the ERR Cause of Death Registry. We calculated standardized mortality ratio using the regional population as the standard.

**Results:**

Among 13,351 patients suffering from MDD, 34,11% died of cancer. In comparison with the ERR population, cancer mortality was 86% (95% CI 1.69–2.04) higher among patients with MDD. Site-specific analyses showed significantly increased mortality for gastrointestinal (esophagus, colon, rectosigmoid, rectum, anus, liver, gallbladder and biliary tract, pancreas), respiratory (trachea, bronchus and lung), uro-genital (breast, ovary and other genital organs, bladder, prostate, kidney, pelvis, ureter and unspecified urinary organs) and other cancers (melanoma, brain and other CNS tumors, lymphoid, hematopoietic and related tissue).

**Conclusions:**

The study confirms the risk for people affected by MDD to have a higher mortality rate and shorter survival in comparison with the general population. These findings highlight the urgent need for greater attention to the physical health of psychiatric patients through optimal collaboration between mental health, oncology and primary care departments.

## Introduction

Disparities and inequalities in the physical health of individuals affected by severe mental illness (SMI), namely major depressive disorders (MDD), schizophrenia, and bipolar disorders, have been widely reported [1–3]. Evidence also suggest a life expectancy 15–20 years shorter and a higher mortality rate from physical disorders among individuals with SMI compared with the general population [4–6].

Cancer is one of the most common physical diseases, with the World Health Organization (WHO) worriedly underlining an increase worldwide by 2050 in terms of incidence (35.3 million new cancer cases from the 2022 estimate of 20 million, 76.6% increase) and deaths (18.5 million cancer deaths by 2050, from the 2022 estimate of 9.7 million, 89.7% increase) [7, 8].

Over the last 20 years, studies have concentrated attention on the relationship of cancer with SMI in terms of disparities, inequities, treatment issues, ethical concerns, and mortality [9–13]. Among SMIs, MDD has been particularly studied. Depression is one of the most prevalent mental disorders in patients with cancer [14, 15] and it is associated with higher mortality compared with cancer patients without depression [16–19]. For example, in a large study of more than 20,000 cancer patients, a diagnosis of major depression (MDD), as evaluated after the diagnosis of cancer, was associated with worse prognosis for all cancers in comparison with non-depressed patients, with similar adjusted hazard ratios regardless of cancer site [20] and with a lower survival time among females [21]. These findings were in part confirmed by studies individuals with pre-existing depression who later developed cancer. A depressive condition or self-reported scores suggesting MDD were associated with higher mortality from cancer in depressed patients followed over time who subsequently developed various forms of cancer [22, 23]. Similar findings were observed among 1,894 men diagnosed with a depressive disorder in the two years prior to a prostate cancer diagnosis, who exhibited worse overall mortality in comparison with men without depression before cancer diagnosis [24]. A large, multicenter prospective study in the United States, also found higher mortality in women who screened positive for depressive symptoms and were later diagnosed with breast cancer [25], although no such association was observed in those who developed colon-rectal cancer [26]. A more recent study showed that women with pre-diagnostic MDD experienced a higher risk of breast cancer mortality than those without prior MDD [27]. There are, however, other studies that did not confirm an increased cancer mortality risk among patients with previous depressive conditions [28, 29] or with MDD [30].

In Italy, no study has examined this field, aside from limited data indicating excess of cancer mortality among patients followed by mental health services for various psychiatric disorders, including common mental disorders, schizophrenia, bipolar disorders, and personality disorders [31–33]. However, cancer-related mortality in individuals specifically diagnosed with MDD has never been examined as a distinct cohort. For these reasons, the aim of this study was to explore cancer mortality rates among all patients followed by mental health departments for MDD compared with the general population.

## Methods

### Study setting and sources of data

This study was carried out in the Emilia Romagna Region (ERR), an area in Northern Italy with approximately 4.5 million inhabitants living across nine provinces (Bologna, Ferrara, Forli-Cesena, Modena, Parma, Piacenza, Ravenna, Reggio-Emilia, and Rimini). In this region the transition from an asylum-based to a community-based psychiatric care system occurred before the nationwide reform introduced with the establishment of the Italian National Health System (law 833, 1978) [34].

For this study, we followed the methodology described elsewhere [32, 33]. In summary, we used the Regional Mental Health Registry to retrieve demographic data (i.e., age, gender, citizenship, and residency), psychiatric diagnosis, and the date of first admission to the mental healthcare system. The Registry includes demographic and clinical data for all in- and outpatients referring to the 13 Departments of Mental Health serving the nine provinces of the ERR. Demographic variables and diagnoses are recorded at the first clinical assessment and subsequently updated by clinicians on a routine basis. In the ERR, all diagnoses are coded using the WHO International Classification of Diseases, 9^th^ Revision, Clinical Modification (ICD-9-CM) [35], as required by the Italian Ministry of Health [36]. For the purposes of the present study, we included only patients aged 18 years or older who had received a diagnosis of MDD (ICD-9 CM codes: 296.2*, 296.3*, 296.90, 296.99, 298.0) from the Mental Health Departments between 1 January 2008 and 31 December 2017. Patients who were not residents of the ERR or who had accessed Mental Health Services prior to 2008 were excluded.

We linked the Mental Health Registry with the Regional Cause of Death Registry using an anonymous identity code. The latter includes all deaths occurring in the Region and contains anonymized information on sociodemographic characteristics, date, setting, circumstances. Causes of death were obtained from the medical section of the death certificate, and classified and coded according to the ICD, tenth revision (Supplementary Table 1), following WHO standards. Both registries undergo systematic quality controls. Change of residence to another Italian region were retrieved from the ERR Registry Office and classified as loss to follow-up, due to the impossibility of obtaining the relevant death certificates.

The ERR Population Archive was used to identify and aggregate data on the age and gender distribution of the general population, which served as the comparator group (https://statistica.regione.emilia-romagna.it/servizi-online/statistica-self-service/popolazione). The study received approval from the local Ethical Committee (N. 341/2019).

### Statistical analyses

To describe the causes of death, we calculated the proportional mortality, defined as the number of deaths within a population due to different causes during a given time period, over the total. In particular, we examined proportional mortality for all cancers and site-specific cancers in both the study population and in the ERR population.

To compare the risk of mortality between the regional and the study population, we computed the standardized mortality ratio (SMR), defined as the ratio of observed deaths in the study population divided by the number of expected deaths, if the study population had the same gender, age class-specific rates as the regional population. Rates for the standard population were obtained from the above-mentioned ERR Cause of Death Registry and the Population Archive. An SMR equal to 1 indicates that observed deaths equal the expected number; values greater than 1 indicate higher-than-expected mortality, whereas values below 1 indicate lower-than-expected mortality. SMRs with 95% confidence intervals (CI) were computed by gender (adjusted for age class only) and for each cancer site with more than 3 observed events. Differences were considered statistically significant when the CI of the SMR excluded the value of 1. The denominator used for rate calculations was person-years, defined as the time from the date of the first access to the ERR Mental Health Departments to 31 December 2017 or to the date of emigration to another region or death (if applicable).

## Results

### Sample characteristics

During the study period, 13,351 patients had at least one access to the ERR mental health departments with a diagnosis of MDD. Table 1 shows the main demographic characteristics of the population at the time of their first psychiatric assessment.

**Table 1.** Demographic characteristics of the study population at their first access to the Mental Health Department Table 1. long description.

		No.	%
Total		13,351	100.00
Gender	Women	8,638	64.70
	Men	4,713	35.30
Age group. Years	18–24	530	3.97
	25–44	3,607	27.02
	45–64	5,525	41.38
	65–74	2,042	15.29
	75–84	1,390	10.41
	85+	257	1.92
Citizenship	Italian	12,053	90.28
	Non italian	1,298	9.72
Residency	Urban^a^	5,061	37.91
	Rural	8,290	62.09

a This includes all residents in municipalities with more than 60,000 inhabitants.

### Proportional mortality from cancer

During the study period, there were 454 deaths from cancer in the study population, and 143,311 in the regional population. The proportion of cancer-related deaths was 34.11% in the study population versus 29.6% in the regional population (*p* < 0.0003) (Table 2).

**Table 2.** Number of all-cause deaths, total cancer deaths, and site-specific cancer death with % proportion over the total, for the regional and the study population, in the whole sample, and stratified by gender Table 2. long description.

	Regional population		Major depression population	
	n.	%	n.	%
**Total deaths (all causes)**	484,102	100.00	1331	100.00
**Total deaths from cancer**	14,3311	29.60	454	34.11
**Site of cancer**				
Esophagus	1,330	0.27	6	0.45
Stomach	9,149	1.89	21	1.58
Colon, rectosigmoid, rectum, anus	15,043	3.11	50	3.76
Liver, gallbladder, biliary tract	9,144	1.89	26	1.95
Pancreas	9,834	2.03	28	2.10
Larynx	996	0.21	2	0.15
Trachea, bronchus and lung	28,492	5.89	98	7.36
Melanoma	1413	0.29	5	0.38
Breast	9,610	1.99	40	3.01
Uterus (cervix, corpus, unspecified)	2,338	0.48	11	0.83
Ovary and other female genital organ	3,087	0.64	13	0.98
Prostate	5,694	1.18	14	1.05
Kidney, pelvis, ureter and unspecified urinary organs	4,420	0.91	16	1.20
Bladder	4,962	1.02	15	1.13
Meninges, brain other parts of CNS	3,460	0.71	14	1.05
Lymphoid, hematopoietic and related tissue	12,032	2.49	45	3.38
**Females ales**				
**Total deaths (all causes)**	255,264	100.00	752	100.00
**Total deaths of cancer**	65,137	25.52	266	35.37
**Site of cancer**				
Esophagus	374	0.15	1	0.13
Stomach	3,949	1.55	13	1.73
Colon, rectosigmoid, rectum, anus	7,095	2.78	36	4.79
Liver, gallbladder, biliary tract	3,738	1.46	11	1.46
Pancreas	5,108	2.00	17	2.26
Larynx	129	0.05	0	0.00
Trachea, bronchus and lung	8,669	3.40	50	6.65
Melanoma	572	0.22	4	0.53
Breast	9,518	3.73	40	5.32
Ovary and other female genital organ	2,338	0.92	11	1.46
Uterus (cervix, corpus, unspecified)	3,087	1.21	13	1.73
Prostate	0	0.00	0	0.00
Kidney, pelvis, ureter and unspecified urinary organs	1,540	0.60	7	0.93
Bladder	1,268	0.50	4	0.53
Meninges, brain other parts of CNS	1,604	0.63	5	0.66
Lymphoid, hematopoietic and related tissue	5,670	2.22	25	3.32
**Males**				
**Total deaths (all causes)**	228,838	100.00	579	100.00
**Total deaths from cancer**	78,174	34.16	188	32.47
**Site of cancer**				
Esophagus	956	0.42	5	0.86
Stomach	5,200	2.27	8	1.38
Colon, rectosigmoid, rectum, anus	7,948	3.47	14	2.42
Liver, gallbladder, biliary tract	5,406	2.36	15	2.59
Pancreas	4,726	2.07	11	1.90
Larynx	867	0.38	2	0.35
Trachea, bronchus, and lung	19,823	8.66	48	8.29
Melanoma	841	0.37	1	0.17
Breast	92	0.04	0	0.00
Ovary and other female genital organ	0	0.00	0	0.00
Uterus (cervix, corpus, unspecified)	0	0.00	0	0.00
Prostate	5,694	2.49	14	2.42
Kidney, pelvis, ureter, and unspecified urinary organs	2,880	1.26	9	1.55
Bladder	3,694	1.61	11	1.90
Meninges, brain other parts of CNS	1,856	0.81	9	1.55
Lymphoid, hematopoietic, and related tissue	6,362	2.78	20	3.45

When examining individual cancer diagnoses, trachea, bronchial, and lung cancers were the most frequent causes of death in both the regional and the study population. Trachea, bronchial, and lung cancers also remained the leading cause of cancer death among female patients with MDD, while in the female population the leading cause of cancer death was breast cancer. In males, lung cancer was always the leading cause.

### Standardized cancer mortality ratio

As reported in Table 3, all-cause mortality was 77% higher in patients with major depression than expected based on the regional population (SMR 1.77, 95% CI 1.67–1.86). Cancer-related mortality was 86% higher than in the regional population (SMR 1.86, 95% CI 1.69–2.04). Females showed a higher excess of mortality due to neoplasms compared with males, although the difference was not statistically significant (Table 3).

**Table 3.** SMR for all causes of deaths, total deaths for cancer, and cancer site in the whole sample and by gender Table 3. long description.

	Observed	Expected	SMR	95% CI	
**Total deaths (all causes)**	1,331	753.15	1.77	1.67	1.86
**Total deaths from cancer**	454	244.53	1.86	1.69	2.04
**Site of cancer**					
Esophagus	6	2.18	2.75	1.01	5.98
Stomach	21	15.02	1.40	0.87	2.14
Colon, rectosigmoid, rectum, anus	50	25.22	1.98	1.47	2.61
Liver, gallbladder, biliary tract	26	15.43	1.69	1.10	2.47
Pancreas	28	17.48	1.60	1.06	2.32
Larynx					
Trachea, bronchus, and lung	98	47.42	2.07	1.68	2.52
Melanoma	5	2.39	2.09	0.68	4.88
Breast	40	20.66	1.94	1.38	2.64
Ovary and other female genital organ	11	5.11	2.15	1.07	3.85
uterus (Cervix, corpus, unspecified)	13	6.99	1.86	0.99	3.18
Prostate	14	7.46	1.88	1.03	3.15
Kidney, pelvis, ureter and unspecified urinary Organs	16	7.07	2.26	1.29	3.68
Bladder	15	7.39	2.03	1.14	3.35
Meninges, brain other parts of CNS	14	6.34	2.21	1.21	3.71
Lymphoid, hematopoietic, and related tissue	45	20.47	2.20	1.60	2.94
**Females**					
**Total deaths (all causes)**	752	451.25	1.67	1.55	1.79
**Total deaths from cancer**	266	136.56	1.95	1.72	2.20
**Site of cancer**					
Esophagus	1	0.81	1.24	0.03	6.90
Stomach	13	7.87	1.65	0.88	2.83
Colon, rectosigmoid, rectum, anus	36	14.29	2.52	1.76	3.49
Liver, gallbladder, biliary tract	11	7.80	1.41	0.70	2.52
Pancreas	17	10.78	1.58	0.92	2.52
Larynx					
Trachea, bronchus, and lung	50	19.47	2.57	1.91	3.38
Melanoma	4	1.22	3.29	0.90	8.42
Breast	40	20.54	1.95	1.39	2.65
Ovary and other female genital organ	11	5.11	2.15	1.07	3.85
Uterus (cervix, corpus, unspecified)	13	6.99	1.86	0.99	3.18
Prostate					
Kidney, pelvis, ureter, and unspecified urinary Organs	7	3.10	2.26	0.91	4.65
Bladder	4	2.44	1.64	0.45	4.21
Meninges, brain other parts of CNS	5	3.67	1.36	0.44	3.18
Lymphoid, hematopoietic, and related tissue	25	11.78	2.12	1.37	3.13
**Males**					
**Total deaths (all causes)**	579	301.90	1.92	1.76	2.08
**Total deaths from cancer**	188	107.97	1.74	1.50	2.01
**Site of cancer**					
Esophagus	5	1.37	3.64	1.18	8.49
Stomach	8	7.16	1.12	0.48	2.20
Colon, rectosigmoid, rectum, anus	14	10.94	1.28	0.70	2.15
Liver, gallbladder, biliary tract	15	7.63	1.97	1.10	3.24
Pancreas	11	6.70	1.64	0.82	2.94
Larynx					
Trachea, bronchus and lung	48	27.94	1.72	1.27	2.28
Melanoma					
Breast					
Ovary and other female genital organ					
Uterus (cervix, corpus, unspecified)					
Prostate	14	7.46	1.88	1.03	3.15
Kidney, pelvis, ureter, and unspecified urinary Organs	9	3.97	2.27	1.04	4.31
Bladder	11	4.95	2.22	1.11	3.97
Meninges, brain other parts of CNS	9	2.66	3.38	1.55	6.41
Lymphoid, hematopoietic, and related tissue	20	8.69	2.30	1.41	3.56

Abbreviations: CI, confidence interval; SMR, standardized mortality ratio.

With regard to site-specific cancer diagnosis, the SMR was >1 for all cancer types. In the overall study population, the excess mortality was significant in all cases except for stomach, ovary, larynx cancer, and melanoma. Among females, a significant excess mortality compared to the regional population was observed for colon, rectum, and anus, trachea, bronchus lung, breast, and uterus cancers, as well as for malignancies of the lymphohematopoietic system. Among males, significantly higher mortality was found for cancers of the esophagus, liver, gallbladder and biliary tract, trachea, bronchus and lung, prostate, kidney, pelvis, ureter and unspecified urinary organs, bladder, and for CNS neoplasms (i.e., meninges, brain and other CNS areas) and for malignancies of the lymphohematopoietic system.

## Discussion

This is the first Italian large study covering a 10-year period and showing that cancer-related mortality in subjects with pre-existing MDD is higher than in the general population. In addition, the study shows an excess mortality for almost all types of cancer, with no differences between males and females. These findings are consistent with studies conducted in the USA, Korea, and China [24, 27], although organizational differences between mental health services in Italy and other countries should be considered.

Results from the present study support the hypothesis that MDD may play a direct or indirect role in cancer pathogenesis and prognosis. More specifically, MDD may be associated with a higher risk of developing cancer, and/or with a higher risk for a worse prognosis. As for a direct role, different possible biological mechanisms underlying MDD may interact with cancer pathophysiology, such as: increased peripheral blood levels of inflammatory cytokines (e.g., interleukin (IL)-1beta, IL-6, tumor necrosis factor and c-reactive protein) [37–39]; activation of the hypothalamic–pituitary–adrenal axis and autonomic nervous system with corresponding deregulation of circadian cortisol rhythm; [40]; alterations of the immune system [41, 42] and of the kynurenine pathways [43]. All these biological phenomena have been documented among patients with MDD and may play a role in cancer [44]. On the other hand, cancer and cancer treatment (e.g., chemotherapy, radiation therapy, immunotherapy) may themselves contribute to impaired neurogenesis, oxidative stress, blood–brain barrier degradation through neuroinflammatory process, and damage to molecular patterns, further reinforcing the relationship between depression and cancer prognosis [45–47]. Another possible explanation is that cancer treatments may be less effective among patients with MDD than among those without depression. Preliminary data suggest that depression is an independent predictor of poorer response to primary chemotherapy in patients with breast cancer, even after controlling for several factors (e.g., age, menopausal status, clinical nodal status, tumor size at diagnosis, estrogen receptor status) [48]. In this regard, it has been suggested that reduced levels of brain-derived neurotrophic factor (BDNF),commonly observed in patients with MDD, may contribute to lower responsiveness to cancer drugs [49]. All these mechanisms may negatively influenceing the course of cancer, ultimately shortening survival.

An indirect role of depression in worsening cancer prognosis is also plausible. A series of studies, reviews, and meta-analyses have shown that patients with SMI, including MDD, are less likely to receive appropriate screening for cervical, breast, colorectal, and prostate cancer [50–53], thereby increasing the risk of receiving a late or advanced-stage diagnosis [54–56]. Also, cancer care may be suboptimal among patients with MDD (e.g., increased risk of receiving non-guideline treatment, neoadjuvant chemotherapy, radiotherapy, or targeted new anti-cancer therapy) [57–61]. In addition, the higher prevalence of smoking, substance abuse, chronic hepatitis B or C infections among individuals with mental illness, including MDD, has been identified as a potential contributor to higher cancer mortality [62].

Finally, evidence shows that depression is one of the factors associated with reduced adherence to several anti-cancer treatments, including adjuvant endocrine therapy and oral chemotherapy [63–65]. This reduction may occur though mechanisms such as low mood, loss of interest, demotivation, negative beliefs about medicines, and abnormal illness behavior [66, 67].

## Strengths and limitations

The major strengths of our study are the large-scale cohort design and the considerable follow-up for the patients attending the MHD, with the possibility to independently and consistently collect data on mental disorders and causes of death. A number of limitations should be mentioned, however. First, we were not ble to distinguish between patients with an underlying diagnosis of MDD and those who were diagnosed after receiving a cancer diagnosis. Although we hypothesize that the percentage of the latter was small, we are aware that this group of patients may be phenomenologically similar but biologically very different. Second, the study did not include the entire regional population of people affected by MDD (e.g., those accessing private psychiatric clinics and/or services or those followed exclusively by their general practitioners). A third limitation is that individuals who were not officially residents of the region were not included in the study. Although the proportion of excluded non-residents was low (about 1% of the caseload), this may theoretically have introduced bias in the estimation of mortality within our cohort. Fourth,despite the fairly long study period, the number of events for some site-specific cancers was very small, resulting in estimates with relatively large confidence intervals and highlighting the need for confirmation in larger studies. Another limitation is that the diagnosis of MDD was categorically based on the ICD-9-CM, without any further specification or sub-typing of the clinical characteristics of depression. [68] Finally, we were unable to examine the multiple variables that could potentially be related to cancer prognosis, as discussed above (e.g., screening for cancer in patients with MDD, timing of cancer development, adherence to anti-cancer therapy, shared biological mechanisms underlying cancer and depression). Therefore, the links between MDD and higher cancer mortality should be explored in more detailed investigations.

## Conclusions

This cohort study showed that patients affected by MDD and followed by ERR departments of mental health have excess mortality from a broad range of site-specific cancers compared to the general population. Further research is needed to investigate the complex relationship between MDD and the prognosis of cancer, as well as the pathophysiological and behavioral mechanisms underlying this association. Given the high number of people worldwideaffected by depression and MDD and, at the same time, the increasing annual incidence of cancer reported by the WHO, the prevention, screening, and treatment of cancer should represent a critical aim for psychiatry [9, 13, 69]. Moreover, closer cooperation between mental health professionals, oncology specialists, and primary care physicians, is essential to facilitate early diagnosis and appropriate treatment among patients who, being primarily affected by MDD, develop cancer [70, 71] and to ensure continuous monitoring of this disorder over time.

## Supporting information

10.1192/j.eurpsy.2025.10048.sm001 Grassi et al. supplementary material Grassi et al. supplementary material

## Data Availability

The datasets generated and/or analyzed during the current study are not publicly available due because of privacy/ethical restrictions, but are available from the corresponding author on reasonable request.

## Abbreviations

CNS: central nervous system
ICD: International Classification of Disease Clinical Modification
WHO: World International Organization

## Supplementary material

The supplementary material for this article can be found at http://doi.org/10.1192/j.eurpsy.2025.10048.

## Long descriptions

### Table 1. Long description

The table presents demographic data for a total population of 13,351 individuals (100.00 percent).

* Gender: Women account for 8,638 (64.70 percent) and Men account for 4,713 (35.30 percent).

* Age group in years:

- 18 to 24: 530 (3.97 percent)

- 25 to 44: 3,607 (27.02 percent)

- 45 to 64: 5,525 (41.38 percent)

- 65 to 74: 2,042 (15.29 percent)

- 75 to 84: 1,390 (10.41 percent)

- 85 plus: 257 (1.92 percent)

* Citizenship: Italian citizens number 12,053 (90.28 percent) and Non-Italian citizens number 1,298 (9.72 percent).

* Residency: Urban residents (defined as municipalities with more than 60,000 inhabitants) number 5,061 (37.91 percent) and Rural residents number 8,290 (62.09 percent).

Navigate back to Table 1..

### Table 2. Long description

The table is organized into three main vertical sections: Whole Sample, Females, and Males. Each section compares the Regional population against the Major depression population using count (n.) and percentage (%).

In the Whole Sample section:

- Total deaths (all causes): Regional 484,102 (100.00%); Major depression 1,331 (100.00%).

- Total deaths from cancer: Regional 143,311 (29.60%); Major depression 454 (34.11%).

- Leading cancer sites for the Major depression population include Trachea, bronchus and lung (7.36%), Colon and rectum (3.76%), and Lymphoid/hematopoietic tissue (3.38%).

In the Females section:

- Total deaths (all causes): Regional 255,264; Major depression 752.

- Total deaths from cancer: Regional 65,137 (25.52%); Major depression 266 (35.37%).

- High-proportion cancer sites for depressed females include Trachea, bronchus and lung (6.65%), Breast (5.32%), and Colon and rectum (4.79%).

In the Males section:

- Total deaths (all causes): Regional 228,838; Major depression 579.

- Total deaths from cancer: Regional 78,174 (34.16%); Major depression 188 (32.47%).

- High-proportion cancer sites for depressed males include Trachea, bronchus and lung (8.29%), Lymphoid/hematopoietic tissue (3.45%), and Liver/biliary tract (2.59%).

Specific cancer sites listed include Esophagus, Stomach, Colon/Rectum, Liver/Gallbladder, Pancreas, Larynx, Trachea/Lung, Melanoma, Breast, Uterus, Ovary, Prostate, Kidney, Bladder, C N S, and Lymphoid tissue.

Navigate back to Table 2..

### Table 3. Long description

The table contains six columns: Observed, Expected, S M R, and a two-column 95% C I. It is divided into three main sections.

1. Whole Sample:

- Total deaths (all causes): Observed 1,331, Expected 753.15, S M R 1.77, C I 1.67 to 1.86.

- Total deaths from cancer: Observed 454, Expected 244.53, S M R 1.86, C I 1.69 to 2.04.

- Selected cancer sites: Esophagus (S M R 2.75), Colon and rectum (S M R 1.98), Trachea, bronchus, and lung (S M R 2.07), Kidney (S M R 2.26), and Lymphoid tissue (S M R 2.20).

2. Females:

- Total deaths (all causes): Observed 752, Expected 451.25, S M R 1.67, C I 1.55 to 1.79.

- Total deaths from cancer: Observed 266, Expected 136.56, S M R 1.95, C I 1.72 to 2.20.

- Selected cancer sites: Colon and rectum (S M R 2.52), Trachea, bronchus, and lung (S M R 2.57), Melanoma (S M R 3.29), and Breast (S M R 1.95).

3. Males:

- Total deaths (all causes): Observed 579, Expected 301.90, S M R 1.92, C I 1.76 to 2.08.

- Total deaths from cancer: Observed 188, Expected 107.97, S M R 1.74, C I 1.50 to 2.01.

- Selected cancer sites: Esophagus (S M R 3.64), Liver and biliary tract (S M R 1.97), Meninges and brain (S M R 3.38), and Lymphoid tissue (S M R 2.30).

Navigate back to Table 3..
