# Supplementary material for: Major depression and mortality from cancer: A 10-year Italian study
Source: Eur Psychiatry. 2025 Dec 4;69(1):e95. doi: 10.1192/j.eurpsy.2025.10048 (PMC13735761; doi:10.1192/j.eurpsy.2025.10048)
Supplement: Supplementary file 1 [file S0924933825100485sup001.docx]

Supplementary Table 1. International Classification of Diseases (ICD)-10 codes for site specific cancers.

|  | **Cancer site** | **ICD-10** |
| --- | --- | --- |
|  |  |  |
| **Total deaths** |  |  |
| **Cancer** |  | C, D1, D2, D3, D4 |
|  | esophagus | C15 |
|  | stomach | C16, D371 |
|  | colon, rectosigmoid, rectum, anus | C18, C19, C20, C21, D374, D375 |
|  | liver, gallbladder, biliary tract | C22 C23, C24 |
|  | pancreas | C25 |
|  | larynx | C32, D380 |
|  | trachea, bronchus and lung | C33, C34, D381 |
|  | melanoma | C43 |
|  | breast | C50, D486 |
|  | ovary and other female genital organ | C56, C57 |
|  | uterus (cervix, corpus, unspecified) | C53, C54, C55 |
|  | prostate | C61 |
|  | kidney, pelvis, ureter and unspecified urinary organs | C64, C65, C66, C68, D410, D411, D412, D413 |
|  | bladder | C67 |
|  | meninges, brain other parts of CNS | C70, C71, C72, D33 |
|  | lymphoid, hematopoietic and related lymphoid lymphopoietic, hematopoietic and related tissue | C81-C96 |
